# Rhythm disturbances as a potential early marker of Parkinson’s disease in idiopathic REM sleep behavior disorder

**DOI:** 10.1002/acn3.50982

**Published:** 2020-02-14

**Authors:** Valérie Cochen De Cock, Delphine de Verbizier, Marie Christine Picot, Loïc Damm, Beatriz Abril, Florence Galtier, Valérie Driss, Cindy Lebrun, Nicolas Pageot, Aurélie Giordano, Chloé Gonzalvez, Pascale Homeyer, Bertrand Carlander, Giovanni Castelnovo, Christian Geny, Benoit Bardy, Simone Dalla Bella

**Affiliations:** ^1^ Sleep and Neurology Department Beau Soleil Clinic Montpellier France; ^2^ Clinical Investigation Centre (CIC) 1411 University Hospital of Montpellier & Inserm Montpellier France; ^3^ EuroMov Université de Montpellier Montpellier France; ^4^ Nuclear Medicine Unit University Hospital of Montpellier Montpellier France; ^5^ Clinical Research & Epidemiology Unit Medical Information Department CHU Montpellier University of Montpellier Montpellier France; ^6^ Sleep Department University Hospital of Nîmes Nîmes France; ^7^ Department of Neurology University Hospital of Montpellier Montpellier France; ^8^ Sleep Department Aubenas Hospital Aubenas France; ^9^ Department of Neurology University Hospital of Nîmes Nîmes France; ^10^ Institut Universitaire de France (IUF) Paris France; ^11^ International Laboratory for Brain, Music, and Sound Research (BRAMS) Montreal Canada; ^12^ Department of Psychology University of Montreal Montreal Canada; ^13^ Department of Cognitive Psychology University of Economics and Human Sciences in Warsaw Warsaw Poland

## Abstract

**Objective:**

We aimed to identify timing distortions in production and perception of rhythmic events in patients with idiopathic REM sleep behavior disorder (iRBD) as early markers of Parkinson's disease (PD).

**Methods:**

Rhythmic skills, clinical characteristics, dysautonomia, depression, and olfaction were compared in 97 participants, including 21 participants with iRBD, 38 patients with PD, and 38 controls, matched for age, gender, and education level. Rhythmic disturbances can be easily detected with dedicated motor tasks via a tablet application. Rhythm production was tested in two conditions: to examine the ability to generate a spontaneous endogenous rhythm, tapping rate and variability in a finger tapping task without external stimulation was measured, while the ability to synchronize to an external rhythm was tested with finger tapping to external auditory cues. Rhythm perception was measured with a task, in which the participants had to detect a deviation from a regular rhythm. Participants with iRBD had dopamine transporter imaging.

**Results:**

Participants with iRBD and PD revealed impaired spontaneous rhythm production and poor rhythm perception compared to controls. Impaired rhythm production was correlated with olfaction deficits, dysautonomia, impaired non‐motor aspects of daily living, and dopamine uptake measures.

**Conclusions:**

Participants with iRBD show impaired rhythm production and perception; this impairment is correlated with other early markers for PD. Testing rhythmic skills with short and inexpensive tests may be promising for screening for potential future PD in iRBD patients.

## Introduction

With the development of neuroprotective drugs for Parkinson’s disease (PD), an early diagnosis of the disease is of paramount importance for minimizing/preventing dopaminergic neuronal loss as soon as possible. At prodromal stage, idiopathic REM sleep behavior disorder (iRBD) seems to be the strongest predictor for synucleinopathies.^1^, ^2^, ^3^, ^4^ This sleep disorder consists in the loss of muscle atonia during REM sleep, allowing patients to enact their dreams during REM sleep. The annual conversion rate from iRBD to an overt neurodegenerative syndrome is 6.3%; this rate raises to 73.5% after 12 years.^5^

In PD, the dysfunctional basal‐ganglia‐cortical circuitry is associated with timing distortions in the perception and production of rhythmic events.^6^, ^7^, ^8^, ^9^ Rhythmic disturbances can be detected in PD with sets of dedicated tests (perceptual and sensorimotor), such as the Battery for the Assessment of Auditory Sensorimotor and Timing Abilities (BAASTA).^10^ BAASTA can be easily administered via a tablet application in patients with PD,^11^ and has proven to be a sensitive and reliable tool.^10^, ^12^ An interesting possibility is that the first signs of a deterioration of basal‐ganglia‐cortical activity at a prodromal stage of PD may already manifest in a subtle but detectable impairment in fine rhythmic skills. We examined this possibility by testing rhythmic skills in patients with iRBD, compared to healthy controls and patients with PD. Other early markers such as olfaction, depressive, dysautonomic, apathic, motor and non‐motor symptoms, as well as dopamine transporter imagery abnormalities were also examined in relation to rhythmic skills.

## Methods

### Participants

Ninety‐seven participants, including 21 iRBD patients (4 females), 38 PD patients (9 females), and 38 healthy controls (9 females), matched for age, gender, and educational level were recruited for the study. Participants with iRBD were recruited at the sleep disorders department of the Beau Soleil Clinic in Montpellier (France). iRBD was confirmed after one‐night polysomnographic recordings in the sleep laboratory according to the criteria of the International Classification of Sleep Disorders, Second Edition,^13^ and confirmed by a sleep specialist (VCDC). None of the iRBD participants had drug‐induced RBD. All of them had a neurological evaluation to rule out the presence of other neurological or neurodegenerative diseases.

Patients with PD were recruited in the Department of Neurology of the Beau Soleil Clinic and the Regional University Hospital of Montpellier (France). Diagnosis was established according to the Queen Square Brain Bank criteria.^14^ All patients were examined by movement disorder specialists (VCDC and CG) and were selected to be at stages 1–3 on the Hoehn and Yahr scale.^15^ Participants were kept on their usual medications during the evaluation. Nineteen (50%) out of 38 patients with PD complained about ongoing RBD.^16^ They did not have polysomnography to confirm RBD; however,  they were considered as probable RBD (pRBD).

Controls were community‐dwelling adults recruited via the volunteers’ database of the Clinical Investigation Centre of the Montpellier University Hospital. They had no history of neurological or psychiatric disorders, no sleep complaint, and displayed no parkinsonian symptoms on physical examination.

The study was approved by the National Ethics Committee (CPP Sud Méditérannée III, Nîmes, France, ID‐RCB: 2013‐A01265‐40). All participants (patients and controls) gave written informed consent to participate.

### Procedure

All participants had a neurological and a psychopathological evaluation, an olfaction assessment, and were tested for rhythmic skills. Participants with iRBD also performed dopamine transporter imaging.

#### Clinical and neuropsychological evaluation

Demographic characteristics and medical history were collected in a preliminary interview. Motor severity of the disease was evaluated on the Hoehn and Yahr scale^15^ and using the revised Movement Disorder Society‐Unified Parkinson's Disease Rating Scale part III (MDS‐UPDRS‐III)^17^ when in “ON” state. The levodopa equivalent daily dose was calculated.^18^ Non‐motor and motor experience of daily living was evaluated using MDS‐UPDRS parts I and II, respectively, and motor complications using part IV.^17^ Dysautonomia was tested using SCOPA‐AUT.^19^

Global cognitive functioning was tested with the Montreal Cognitive Assessment.^20^ Depressive symptoms were evaluated using the Beck Depression Inventory,^21^ and apathy using the Lille Apathy Rating Scale.^22^

#### Rhythmic skills

Participants’ rhythmic abilities were measured with a subset of tasks taken from the Battery for the Assessment of Auditory Sensorimotor and Timing Abilities (BAASTA).^10^ The battery has proven sensitive to rhythm deficits in a variety of conditions including PD.^7^, ^23^, ^24^, ^25^ The tests lasted 15–20 min and no special training was needed.

#### Rhythm production

Rhythm production was examined with two tapping tests. Spontaneous endogenous rhythm generation was tested with an *unpaced finger tapping task* in which participants were asked to tap for 60 sec at their most comfortable rate with their dominant hand and with their non‐dominant hand in separate trials. Note that participants were not asked to tap as fast as possible and that this task is not a measure of akinesia. To measure synchronization to an external rhythm, a *paced finger tapping test* was performed. Participants tapped with (1) the sounds of a metronome and (2) to the beat of short musical excerpts. Each metronome sequence was formed by 60 piano tones (tone frequency = 1319 Hz) with an inter‐stimulus interval of 600 msec. The musical stimuli were two short musical excerpts (from Bach's “Badinerie,” and from Rossini's “William Tell Ouverture”) with a salient beat. Each trial in both tests was repeated twice. Taps were recorded using a digital drum pad (Roland HPD‐20) and stimuli were delivered via headphones (Shure SRH940).

For unpaced tapping, tapping rate (mean inter‐tap interval [ITI]) and variability (coefficient of variation of the ITI: standard deviation (SD) of the ITI/mean ITI) were calculated. For paced tapping, tapping variability was computed as done for unpaced tapping.

#### Rhythm and duration perception

The *duration discrimination test* measures the perception of duration. Participants were presented with pairs of pure tones (frequency = 1 kHz; interval between tones = 600 msec). The first tone lasts 600 msec (standard duration) while the second tone (comparison) varies between 600 and 1000 msec. The task was to assess whether the comparison tone lasted longer or not than the standard tone.

The *anisochrony detection test with tones* assesses the sensitivity to an irregularity (i.e., a time shift) in a rhythmic sequence of isochronous stimuli. Participants listened to sequences of five tones (tone frequency = 1047 Hz, tone duration = 150 msec, inter‐onset interval – IOI: 600 msec). When present, the time shift occurred on the fourth tone, which was presented earlier than expected based on the IOI of the preceding tones. The magnitude of the time shift could reach 30% of the IOI (180 msec). The task was to judge whether the rhythm of each sequence was “regular” or “irregular.”

The *anisochrony detection test with music* assessed participants’ ability to detect a time shift (i.e., deviant beat) in a short musical excerpt, taken from the same stimuli used in the paced tapping test. When present, the time shift occurred on the fifth beat, and had the same magnitude as in the anisochrony detection test with tones. The task was to judge whether the rhythm of each excerpt was “regular” or “irregular.”

In these three tasks, we calculated a perceptual threshold (% of the standard duration in duration discrimination, or of the IOI in anisochrony detection) using an adaptive method.^10^

#### Dopamine transporter imaging

##### SPECT protocol

Each patient with iRBD received a single intravenous administration of 110MBq of 123I‐Ioflupane (123I‐FP‐CIT, Dat SCAN; GE Healthcare) which binds to the presynaptic dopamine transporter receptors in the membranes of the nigrostriatal nerve terminals. To block thyroid uptake of free radioactive iodine, potassium perchlorate was administered at least 60 min before and 24 h after the radiopharmaceutical injection. Imaging was performed 4 h after 123I‐FP‐CIT using a dual‐head gamma camera (Hawekeye; General Electric Medical Systems), equipped with low‐energy high‐resolution collimators (LEHR). Single photon emission computed tomography (SPECT) studies were acquired using the following parameters: 128 × 128 matrix, zoom 1.28, 120 projections (radius of rotation at 15 cm, a circular orbit), 30 sec per projection, energy window 20% over 159 keV. The slice thickness was 3.45 mm. Reconstruction was performed by filtered back projection with a Butterworth filter (cutoff frequency 0.5, order 10) to produce transaxial slices, reoriented along the fronto‐occipital axis.

##### SPECT analysis

For the semi‐quantitative analysis of 123I‐FP‐CIT striatal uptake, the ratio of specific to nonspecific binding was calculated by summing the three adjacent transverse slices that showed the most intense striatal uptake. A standard region of interest (ROI) template, including fixed regions for the putamen, caudate nuclei and occipital cortices, was placed on the summed images. Therefore, the same ROIs were used in all patients for both images. The ratio of specific to nonspecific binding was then calculated as 123I‐FP‐CIT binding = (ROI‐O)/O, in which the ROI represents the mean counts in putamen or caudate nucleus and O represents the mean counts in the occipital cortex. Every scan was normalized to a 123I‐FP‐CIT template obtained from the 21 healthy controls, matched for age and sex. As reported by others,^26^ we calculated the caudate‐to‐putamen ratio as the maximum caudate uptake divided by the maximum putamen uptake. The putamen asymmetry index was calculated as the ratio between the minimum and the maximum putamen uptake. The specific striatal‐to‐occipital cortex uptake ratios, caudate‐to‐putamen ratio, and putamen asymmetry index were regarded as abnormal when the values were less than two SDs of the mean of the values measured in controls.

Abnormal DAT imaging was determined when at least one of the measures evaluated was abnormal.

#### Olfaction

The olfactory function was tested by asking participants if they reported a subjective olfactory dysfunction, and by administering them three separate subtests using standardized Sniffin’ Sticks^27^ to examine their olfactory threshold, odor discrimination and odor identification. The global olfactory function was categorized into five stages using the sum of threshold, discrimination and identification scores as anosmia (sum score < 15), severe hyposmia (15 < score <20), moderate hyposmia (20 < score < 25), mild hyposmia (25 < sum score < 30), and normosmia (30 < sum score).^28^

### Statistical analyses

Categorical variables were presented as percentages and quantitative variables as means and SDs. Groups were compared on the results of clinical tests, rhythm tests, dopamine transporter imaging, and olfaction using independent‐sample t tests, or Mann–Whitney tests, depending on the normality of the distribution for continuous variables. Chi‐squared tests or Fisher’s exact tests were used for categorical ones. Pearson correlation coefficient was calculated for continuous variables between rhythmic performances and other early markers for PD, such as motor signs, olfaction, dysautonomia, cognition, and DAT fixation. Significance level was set at *P* < 0.05.

## Results

Demographic and clinical characteristics for participants with iRBD, PD, and controls are presented in Table 1.

**Table 1:** Clinical characteristics of patients with idiopathic REM sleep behavior disorder (iRBD), Parkinson’s disease (PD), and controls.

	Controls	iRBD	PD	*P*	
	(*n* = 38)	(*n* = 21)	(*n* = 38)	iRBD versus Controls	iRBD versus PD
Age (years)	69.1 ± 7.2	68.7 ± 6.9	69.1 ± 7.7	0.8	0.8
Sex (% of men)	81.6	81.0	81.6	1.0	1.0
Body mass index (kg/m^2^)	26.0 ± 2.2	26.1 ± 3.7	25.3 ± 3.7	0.9	0.5
Education (years)	12.9 ± 3.7	12.5 ± 4.1	13.4 ± 4.0	0.7	0.4
Motor aspects of experiences of Daily Living (MDS‐UPDRS‐II/52)	0.5 ± 1.1	1.1 ± 1.9	9.9 ± 6.2	0.07	<0.0001
Motor disability (MDS‐UPDRS‐III/132) when treated for PD	3.8 ± 3.0	5.3 ± 4.5	20.5 ± 11.6	0.08	<0.0001
Axial signs	0.5 ± 0.6	1.0 ± 0.9	2.9 ± 2.1	<0.05	<0.0001
Tremor	0.0 ± 0.0	0.7 ± 0.9	2.8 ± 2.6	<0.01	<0.001
Dysautonomia (SCOPA‐AUT)	5.4 ± 5.6	8.2 ± 9.2	14.0 ± 11.0	<0.05	<0.01
Non‐motor aspects of experiences of Daily Living (MDS‐UPDRS‐1A + 1B)/52)	3.8 ± 3.2	6.8 ± 4.9	11.6 ± 5.8	<0.01	<0.001
Global cognitive function (MoCA)	27.3 ± 1.9	26.2 ± 3.3	26.4 ± 2.8	0.2	0.5
Apathy (LARS)	−10.9 ± 2.8	−9.3 ± 2.9	−8.8 ± 3.1	*	*
Depression (Beck Depression Inventory)	5.5 ± 4.9	8.3 ± 7.1	13.4 ± 9.8	*	<0.05

Data are mean ± SD.

* Scores within the normal range justifying no statistical comparison.

Patients with PD had a mild disease with a mean Hoehn and Yahr at 1.7 ± 0.7. Disease duration was 6.8 ± 4.5 years and age at onset 62.2 ± 8.8 years. Motor complications were limited (MDS‐UPDRS‐IV at 1.7 ± 2.6/24). Levodopa equivalent daily dose was 258.7 ± 214.7 mg and 47.6% of the patients received dopamine agonists and the same proportion received monoamine oxydase inhibitors.

In participants with iRBD, subtle motor manifestations were common, with significantly more tremor and axial signs on motor examination, more non‐motor complaints especially concerning sleep as expected (MDS‐UPDRS‐1A + 1B) and more dysautonomia (Table 1).

RBD duration was not different between patients with iRBD and patients with PD and pRBD (11.4 ± 11.2 vs 12.2 ± 12.4, *P =* 0.7).

As expected, participants with iRBD had significantly less motor and non‐motor, dysautonomic and depressive symptoms than patients with PD.

### Rhythmic skills

#### Rhythm production

**Figure 1:**
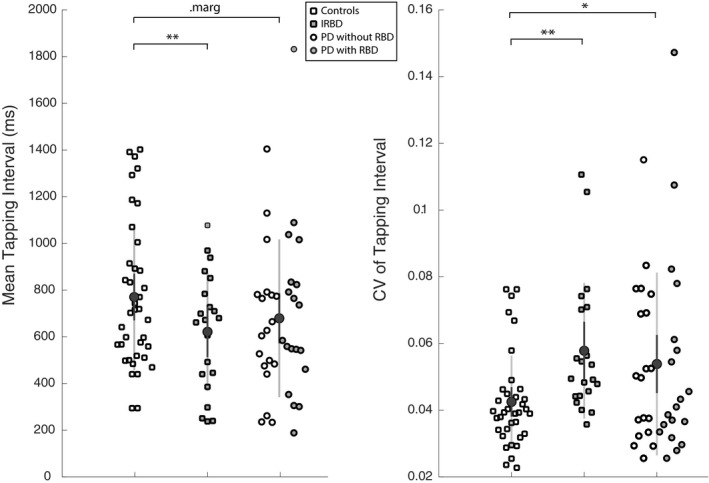
Individual spontaneous tapping rate and variability in participants with iRBD, healthy controls and patients with PD with and without probable RBD. The light grey and dark grey lines, respectively, lay over a 1.96 SEM (95% confidence interval) and 1 SD.

Results from unpaced tapping (dominant hand) and its variability are reported in Figure 1. Participants with iRBD tapped faster and showed greater variability than controls. They did not differ from patients with PD.

Similarly, in paced tapping participants with iRBD tapped with greater variability than controls (coefficient of variation of the inter‐tap interval: 0.073 ± 0.049 vs. 0.048 ± 0.019, *P* = 0.02), and did not differ significantly from patients with PD (0.053 ± 0.045, *P* = 0.1).

#### Rhythm and duration perception

Participants with iRBD performed poorer than controls in detecting a rhythmic irregularity in a complex rhythm (anisochrony detection with music, threshold 16.9% ± 9.4 vs. 12.3% ± 7.3, *P* = 0.04). This difference was not visible with a simpler rhythm (anisochrony detection with tones, 12.8% ± 5.1 vs. 12.3% ± 5.0, *P* = 0.6) and in discriminating simple durations (21.0% ± 10.4 vs. 21.4% ± 8.3, *P* = 0.3). Patients with PD and controls did not differ on any of the perceptual tasks: duration discrimination (24.7% ± 11.8, *P* = 0.2) and anisochrony detection (with tones: 13.4% ± 6.1, *P* = 0.4; with music: 12.5% ± 6.5, *P* = 0.4). Patients with PD with and without pRBD did not differ on these perceptual measures (*P*s > 0.55).

### Dopamine transporter imaging

Comparing IRB and controls, we observed that fixation indexes were reduced for left putamen (1.35 ± 0.37 vs. 1.74 ± 0.35; *P* < 0.001), right putamen (1.37 ± 0.41 vs. 1.78 ± 0.33; *P* < 0.001), left caudate (1.73 ± 0.41 vs. 2.13 ± 0.38; *P* = 0.001), right caudate (1.71 ± 0.38 vs. 2.01 ± 0.34; *P* = 0.005), and putamen asymmetry (0.87 ± 0.13 vs. 0.92 ± 0.05; *P* = 0.03), and increased for caudate/putamen ratio (1.28 ± 0.25 vs. 1.18 ± 0.12; *P* = 0.05). However, among the 21 participants with iRBD, only 9 had abnormal DAT scans results.

### Olfaction

Subjectively, 55% of the patients with iRBD complained about olfaction dysfunction. This was significantly more frequent than in controls (21%, *P* = 0.03) but less than in patients with PD (100%, *P* = 0.001). Olfaction threshold, discrimination, identification, and sum scores were lower in participants with iRBD than controls (respectively, 3.2 ± 2.1 vs. 7.4 ± 1.7, *P <* 0.0001; 8.6 ± 3.2 vs. 11.4 ± 2.0, *P* = 0.001; 7.6 ± 3.2 vs. 13.1 ± 1.6, *P* < 0.0001; 19.4 ± 7.1 vs. 31.9 ± 3.4, *P* < 0.0001) and were not different than in PD (respectively in the same order for PD 3.7 ± 1.5, 7.8 ± 3.4, 6.8 ± 3.6, and 18.2 ± 7.0, all values the four values of *P*> 0.4). Moderate, severe hyposmia, and anosmia were present in 75% of the participants with iRBD and PD but not in controls (*P* = 0.0001) who all had normosmia or mild hyposmia. In this study, the sensitivity of olfaction dysfunction (moderate, severe, or anosmia) to be associated with iRBD was 75% and specificity was 100%.

### Correlations between rhythmic performances and typical early markers of PD

**Figure 2:**
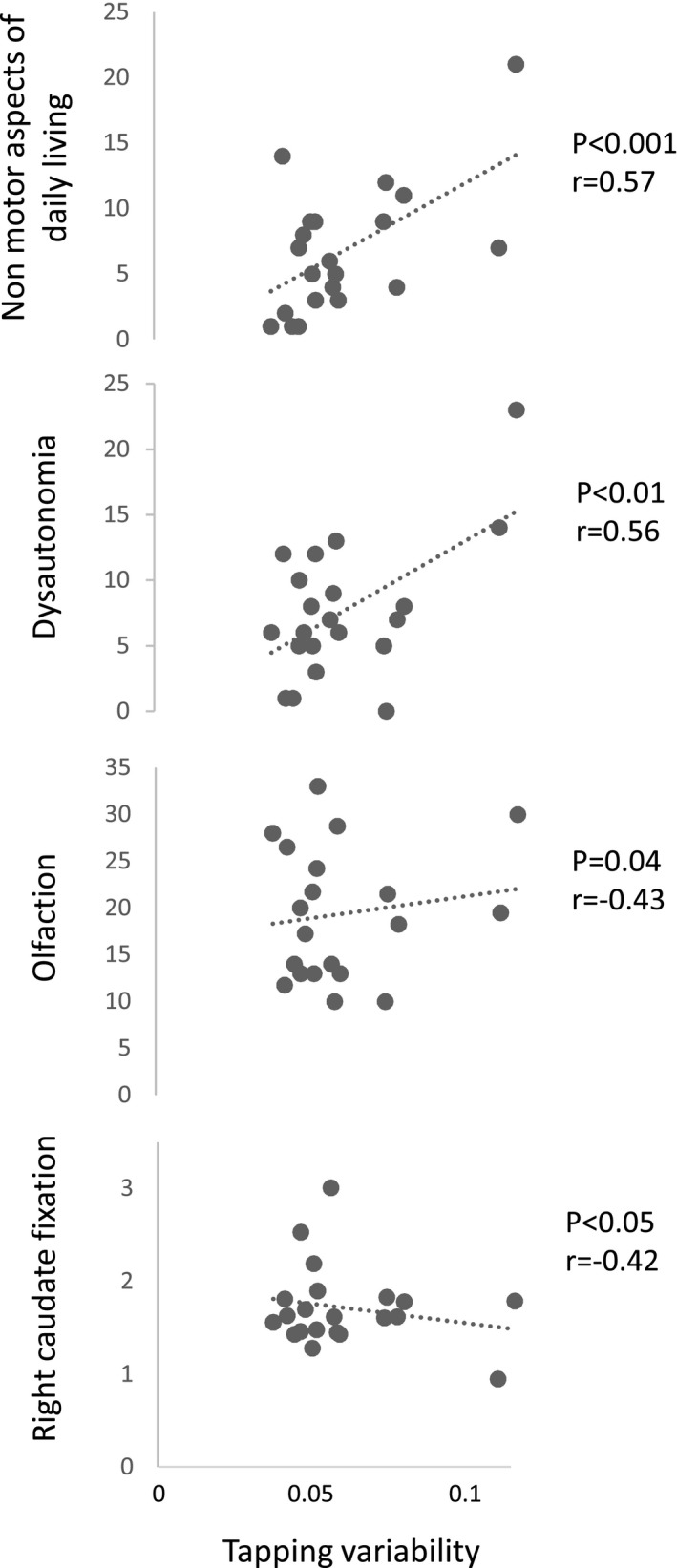
Correlations between the variability of spontaneous tapping and the different markers of future PD (non‐motor symptoms, dysautonomia, olfaction dysfunction, right caudate dopamine fixation reduction) among patients with iRBD.

In the iRBD group, spontaneous tapping variability was correlated with non‐motor aspects of daily living, dysautonomia, olfaction deficits, and right caudate dopamine fixation ratio (Fig. 2). Paced tapping variability only correlated with odor discrimination (Pearson’s correlation coefficient 0.53; *P* = 0.02). We did not identify significant correlations between anisochrony detection with music and the typical early markers of PD.

## Discussion

The goal of this study was to examine whether an impairment of rhythm perception or production can precede full‐blown PD and whether this information can be used to predict potential future PD in iRBD. We tested in a population of patients with iRBD, a risk group for future PD, if deficits of rhythm perception or production can act as an early marker of PD and whether individual differences in these abilities are related to other well‐known early markers of PD, such as motor and non‐motor aspects of daily living, dysautonomia, olfaction dysfunction, and DAT abnormalities. We found that the production of spontaneous endogenous rhythm in iRBD, tested with finger tapping, was faster and more variable than in controls. Patients with iRBD did not differ from patients with PD on these measures. Instead of reducing speed because of akinesia, patients with both PD and iRBD tapped faster and less regularly than controls showing an impairment in beat generation present at the earliest stages of the disease. Moreover, we found signs of poorer rhythm perception (with a complex rhythmic sequence) in iRBD relative to controls. The variability in producing a spontaneous rhythm in iRBD was correlated with other predictors of synucleinopathies such as non‐motor aspects of daily living, dysautonomia, olfaction, and right caudate dopamine fixation. The variability in producing a paced rhythm was correlated with odor discrimination.

There is consistent evidence that rhythm perception and production can be deteriorated in PD probably as a result of the dysfunctional basal‐ganglia‐cortical circuitry characteristic of the disease.^6^, ^7^, ^8^, ^9^ Variability in rhythmic abilities in PD can explain why, in most of the cases, a compensation provided by an external rhythmic auditory cue can improve patients’ motor performance, such as gait.^29^

An intriguing question is as to whether rhythmic deficits can manifest earlier than the appearance of PD cardinal motor symptoms. iRBD is a natural candidate to address this question, as it is a condition leading to synucleinopathies within 12 years in more than 70% of the cases. For the first time here we show evidence that degraded rhythmic abilities might act as behavioral markers of the evolution of neurodegeneration in the basal‐ganglia‐cortical circuitry while compensatory networks (probably cerebello‐cortical circuits) take over.

Different studies are ongoing on neuroprotective drugs in PD and hopefully one will soon demonstrate their efficacy. Motor symptoms of PD appear when more than 50–80% of the dopaminergic neurons are destroyed. The effect of neuroprotective drugs used at this stage of the disease is likely to be limited by the already advanced neuronal loss. Earlier intervention, thanks to prodromal markers of the disease, has in theory far more potential for slowing down the incumbent neurodegeneration. Deficits in rhythm perception and production can be easily detected with tests implemented in a tablet application (e.g., BAASTA).^10^, ^11^, ^30^ This method is not invasive, cost‐effective, fast to administer, and can be made easily available. It could be a first‐line screening for PD, associated with other first‐line motor testing applications^31^ and complemented at a second time by other markers and especially dopamine transporter imaging, a specific but more expensive method.

Our study identified deficits in rhythm abilities as potential early markers for PD, correlated with other markers of future PD in iRBD. However, not all of them were probed, and especially color vision was not examined. Our SPECT results need further validation since their reference group was different from our control group in the study. Also, rhythm production and perception were impaired in patients for whom other markers of future PD were present but we do not know yet if they will develop with this disease. Only a prospective follow‐up study will allow us to confirm this hypothesis. This study could also allow examining the evolution with time of the different perception and production parameters of rhythmic skills with neurodegeneration and compensatory networks.

Rhythmic skills and especially rhythm generation seem to be a promising early marker for future PD. Further large studies are needed to characterize more precisely their sensitivity and specificity to detect ongoing hidden synucleinopathies and among them to identify possible differences. Prospective follow‐up of these dysfunctions could also allow better understanding of the networks that support them and how they adapt as neurodegeneration goes on. Finally, this intriguing finding of rhythm disturbances in iRBD could be a new abnormal biomarker that could complete the cluster of other markers actually utilized to predict imminent phenoconversion to overt PD, or other synucleinopathies, within 3 or 4 years, and then to realistically test promising neuroprotective agents.

## Conflict of Interest

None to declare.
